# Bibliometric Analysis of Studies on Coffee/Caffeine and Sport

**DOI:** 10.3390/nu13093234

**Published:** 2021-09-17

**Authors:** Nicolás Contreras-Barraza, Héctor Madrid-Casaca, Guido Salazar-Sepúlveda, Miguel Ángel Garcia-Gordillo, José C. Adsuar, Alejandro Vega-Muñoz

**Affiliations:** 1 Facultad de Economía y Negocios, Universidad Andres Bello, Viña del Mar 2531015, Chile; nicolas.contreras@unab.cl; 2 Facultad de Ciencias Económicas, Administrativas y Contables, Universidad Nacional Autónoma de Honduras, Tegucigalpa 11101, Honduras; hector.madrid@unah.edu.hn; 3 Departamento de Ingeniería Industrial, Facultad de Ingeniería, Universidad Católica de la Santísima Concepción, Concepción 4090541, Chile; gsalazar@ucsc.cl; 4 Public Policy Observatory, Universidad Autónoma de Chile, Santiago 7500912, Chile; miguel.garcia@uautonoma.cl; 5 Promoting a Healthy Society Research Group (PHeSO), Faculty of Sport Sciences, University of Extremadura, 10003 Cáceres, Spain; jadssal@unex.es

**Keywords:** drinkable nutrients, coffee consumption, caffeine effects, metabolism, sports performance, risk factors, sports health, energy drinks, sugar-sweetened beverages, bibliometrics

## Abstract

This article provides an empirical overview of coffee/caffeine studies in relation to sport worldwide, an incipient but growing relationship that has existed since 1938, although systematized over time since 1999. The extracted articles were examined using a bibliometric approach based on data from 160 records stored in the Web of Science (JCR) between 1938 and August 2021, applying traditional bibliometric laws and using VOSviewer for data and metadata processing. Among the results, these articles highlight an exponential increase in scientific production in the last two decades, with a concentration in only 12 specific journals, the hegemony of the USA among the co-authorship networks of worldwide relevance, and the thematic and temporal segregation of the concepts under study. This article concludes a high fragmentation of the authors with the highest level of scientific production and an evolution of almost 20 years in relevant thematic topics, and a concurrent concentration in three large blocks: (1) coffee consumption and risk factors, (2) health and coffee consumption, and (3) metabolism and sport correlated with the intake of coffee, which are distanced in time, providing evidence of an evolution that gives way to the irruption of alternative visions in the relationship of coffee and caffeine with sport.

## 1. Introduction

Through a bibliometric analysis, this work aimed to analyze the interest and scientific evolution of the caffeine effect in both general and specific populations. Caffeine intake is widespread among the general population. Beyond being a central nervous system stimulant [1], caffeine is consumed in many countries as a cultural activity. Increasingly, however, its intake has motivations related to sports performance. In this sense, many studies have shown remarkable results for caffeine as an ergogenic supplement [2,3]. On the other hand, there is also evidence of risks associated with caffeine in some population groups, such as pregnant women, when consumed in excess [4]. In older people, caffeine intake is associated with a slight increase in blood pressure and risk of some cardiovascular diseases; however, it is associated with a lower risk of overall mortality [5]. Although the effects of caffeine impact a multitude of populations and subject areas, not all the above classifications have generated the same interest in the scientific community. Thus, upon further and more detailed analysis, three main categories were observed: (1) occupational development (physical and/or cognitive performance) [6], (2) nutrition (thermogenic activity) [7], and (3) biological (sleep biology, etc.) [8].

### 1.1. Supply and Impact of Caffeine on the Human Body

Caffeine is an alkaloid compound of xanthine, which is commonly and daily consumed by people and present in different beverages such as tea, coffee, sodas, energy drinks and some medicines [9,10,11].

Its consumption has been experimented with through chewing gum, mouthwash, and oral and nasal aerosols, which mainly affects brain stimulation and brain connections, triggering excitement and alertness and improving mood [12]. It is used as a medication in apnea for infants, in pain relief therapy, and in the short-term treatment of fatigue symptoms [13].

The effects of caffeine use have been associated with an improvement in muscular endurance and contractile performance, and in the synthesis of nitric oxide, which positively impacts blood flow and progression among patients with Parkinson’s disease [14,15,16,17]. With a continuous and high consumption of caffeine, this negatively affects human functioning due to the increase in blood pressure, increasing nervousness, euphoria, irritability, insomnia, and diuresis. [18,19].

The combination of thermogenic ingredients enables energy metabolism, weight control, and sports performance without causing negative side effects [7]. Due to mild elevations in blood pressure, caution should be exercised in those at increased risk for hypertension or prehypertension [10,20]. Taken daily, thermogenic dietary supplementation may increase total energy expenditure, which may lead to reductions in fat mass over time [20].

### 1.2. The Effects of Caffeine and Sports Performance

Many activities require optimal physical and cognitive function to ensure success, safety, and productivity in the workplace (armed forces, first responders, carriers, and factory workers). [6] In circumstances of restricted sleep, frequent caffeine intake is effective in maintaining physical and cognitive abilities [6,21].

Sports activity also requires these strategies, with caffeine being frequently consumed in sports and physical activity for its ergogenic properties, such as stimulation of the central nervous system and a greater development of muscular strength [18,22,23,24,25,26,27].

Several investigations indicate that coffee consumption could have a positive impact on physical performance and sports skills [6,21,25,26,28]. However, the dose-dependent influence induced by caffeine on discipline-specific performance varies [29,30]. An example is that evidence from thirteen out of seventeen studies indicated the effects of different magnitudes on various physical activities and cognitive abilities, including endurance capacity, weightlifting performance, simple reaction time, and memory [22].

Fett et al. [31] found that caffeine improved fatigue tolerance and strength in young women, being useful for improving performance in women who practice sports and physical activities [31]. One systematic review study concluded that they had found no significant differences between sexes in terms of the effect of caffeine supplementation on aerobic performance and the fatigue index. However, four studies of seven articles (57.1%) showed that the ergogenicity of caffeine for anaerobic performance was greater in men than in women [3]. Despite the importance of eccentric contractions in athletic performance, we have not identified research evaluating the ergogenic effects of caffeine in this type of cantilever exercise [32]. Supplementation of 6 mg caffeine per kg can be considered to maximize physical performance in sports with high endurance demands [32].

Although there are several ways to consume caffeine during exercise, (caffeine anhydrous, sports drinks, caffeinated carbohydrate gels, and chewing gum), a popular method among athletes is coffee, which is also used by sportsmen [25,26,28]. There are several studies in different disciplines on the effect of caffeine. One study was conducted to examine the effect of the single ingestion of 3, 6, or 9 mg/kg body weight of caffeine and placebo (PLA) on specific performance in judo sparring and sparring activities [29]. Another study in cyclists discovered that caffeine before an exercise session can provide ergogenic effects on anaerobic performance, especially in trained athletes [33]. Another study aimed to evaluate the acute effects of caffeine ingestion on reactive agility performance in soccer players [27]. A different study investigated the effect of a caffeinated energy drink on various aspects of performance in sprint swimmers [18].

Thus, the temporal evolution of coffee/caffeine studies in relation to sport and the irruption of new paradigms around its consumption makes it necessary to conduct a broad and updated meta-analytical study that provides a panoramic vision to the scientific and practice communities, which is feasible through a bibliometric approach that analyzes data and metadata from pre-existing specialized articles.

## 2. Materials and Methods

We used a set of articles as a homogeneous basis for citation, counting the main col-lection of Web of Science (WoS) [34], by selecting articles published in WoS-indexed journals in the Science Citation Index (WoS-SCI) and Social Science Citation Index (WoS-SSCI), based on a search vector [35] about coffee and sport (TS = coffe* and sport*) and without restricted time parameters, performing the extraction on 4 August 2021.

The resulting set of articles was analyzed bibliometrically, a meta-analytic method [36] previously used in nutrition and dietetics journals for analyzing a general bibliometric [37,38,39,40], scientific production [41,42,43], science mapping [44,45,46], and scientific trends [44,46,47,48] in terms of their exponential growth, to ensure a critical mass of documented scientific production that ensures interest in the international scientific community and gives meaning to the subsequent analysis [49,50], determining the time median and its contemporary and obsolete periods. In terms of concentrations, Bradford’s law of concentrations was applied to the journals, fragmented into thirds of articles, avoiding the exponential decrease in decreasing performance by expanding the search of references in scientific journals peripheral to the topic under study [51,52]. Lotka’s law about authors was applied to identify the most prolific group of authors and study them in isolation from the other authors with a smaller number of articles based on the unequally distributed scientific production among authors [53]. The Hirsch index or h-index was used for articles based on the set of articles most cited by the scientific community and the citations they have received in other publications of the WoS core collection, established as the “n” documents cited “n” times or more [54,55]. Zipf’s law on words was applied to empirically determine words with the highest frequency of occurrence in the set of articles studied (author keywords, keywords plus, or key terms on titles or abstract) [56]. Information processing and the visualization of spatiality, co-authorship, and co-occurrence [57,58] were processed with VOSviewer Software, using fragmentation analysis with thematic and time trend visualization outputs [59,60].

## 3. Results

**Figure 1:**
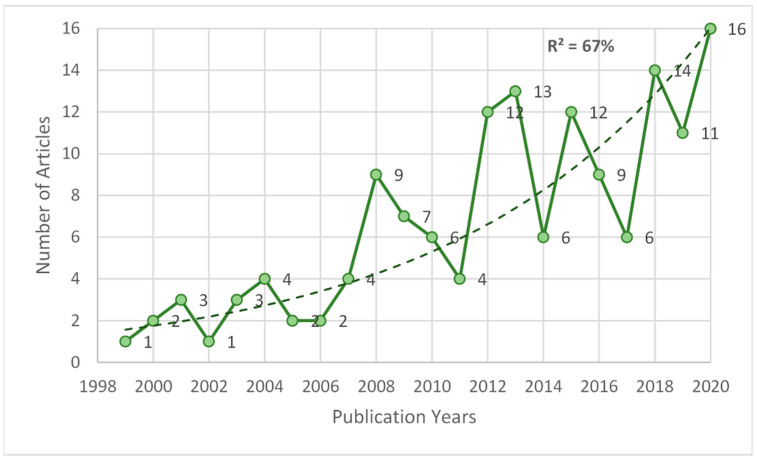
Temporary trend of publications on coffee/caffeine and sport (1999–2020).

The extraction achieved comprised 160 articles between 1938 and 2021 (Table S1), including publication advances (empty year data, assigned to the year of advance). However, only between 1999 and 2020 is there a continuity of publications, and it is possible to check the adjustment to exponential growth (in this case 67%) with a total of 147 articles in this period (See Figure 1).

The % fit (R^2^) is interpreted under the normal ranges of a fit to the data. Furthermore, by splitting the number of articles with the median into two halves, the newer half of publications are the contemporary articles (2014–2020) and the other older half, the obsolete articles (1999–2013), except for the contemporary articles (information that will be supplemented later with the h-index).

When trying to establish the Bradford zones, selecting the journals and articles published in them, we can observe that there is no distinct level of publication that serves as a criterion to divide the set of journals into three thirds. In this case, we opted for an elitist criterion, understanding that there is a core of 12 journals that contain 30% of the articles, followed by seven journals that have published two articles on this topic and another 98 journals that only have one article. Therefore, we have a weak core and a high peripheral dispersion. Additionally, when reviewing the temporality of these publications, for the main journals in time periods, we see how the number of articles in the Bradford core journals has doubled in the last decade (See Table 1).

**Figure 2:**
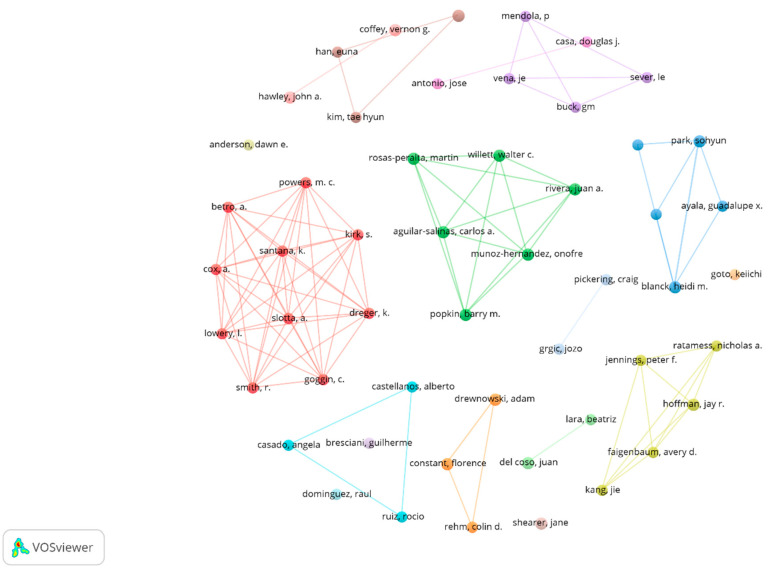
Co-authors graph on coffee/caffeine and sport (only prolific authors).

These 160 articles are the result of the scientific production of 667 authors, so it is estimated that the number of the most prolific authors is 26 (Root Square (667) ≈26). However, in empirical terms, there are 52 authors with two articles and 16 authors with three articles. When exploring with VOSviewer for the total of 52 authors, not all of them relate to others. The graph of 17 clusters shown in Figure 2 was obtained by means of a normalization analysis with the fractionation method (attraction: nine; repulsion: one).

**Figure 3:**
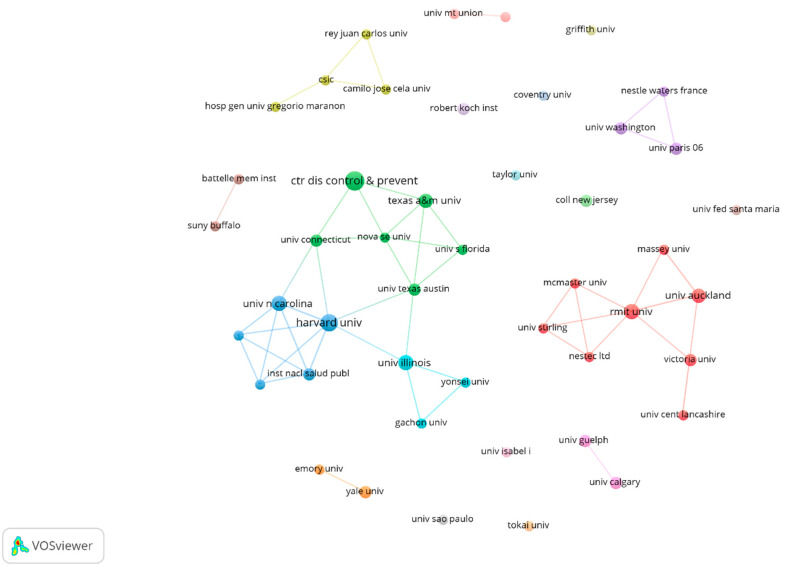
Organizational co-authors graph on coffee/caffeine and sport (only prolific organization authors).

The fragmentation level is reinforced at the organization level. The graph in Figure 3 shows 46 organizations with a minimum participation of two articles, for a total of 276 organizations (there are 230 organizations with only one contribution on coffee/caffeine and sport studies). This strengthens the identification of an actor’s configuration, based on competitive interaction or structural equivalence [61,62,63].

**Figure 4:**
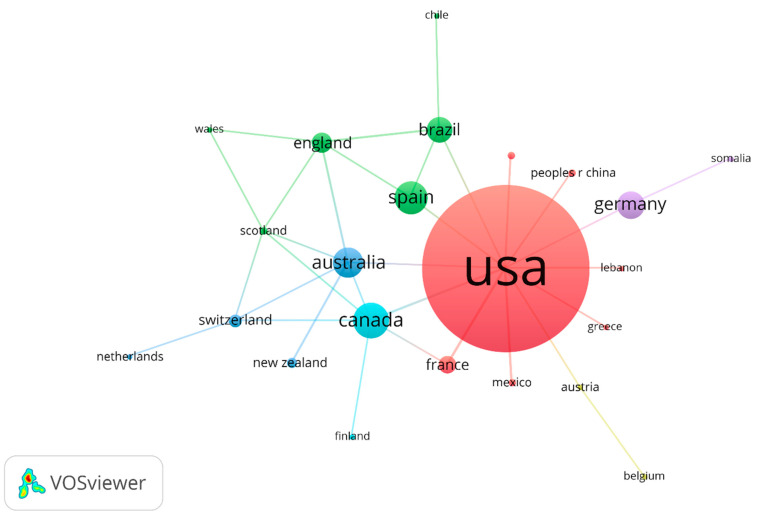
Country/region co-authors graph on coffee/caffeine and sport (only prolific organization authors).

Finally, in terms of co-authorship at the country or region level, the USA stands out from the rest of the countries (33 countries) as a hegemony in the production of knowledge on coffee/caffeine and sport with 65 contributions per author affiliation (See Figure 4).

**Figure 5:**
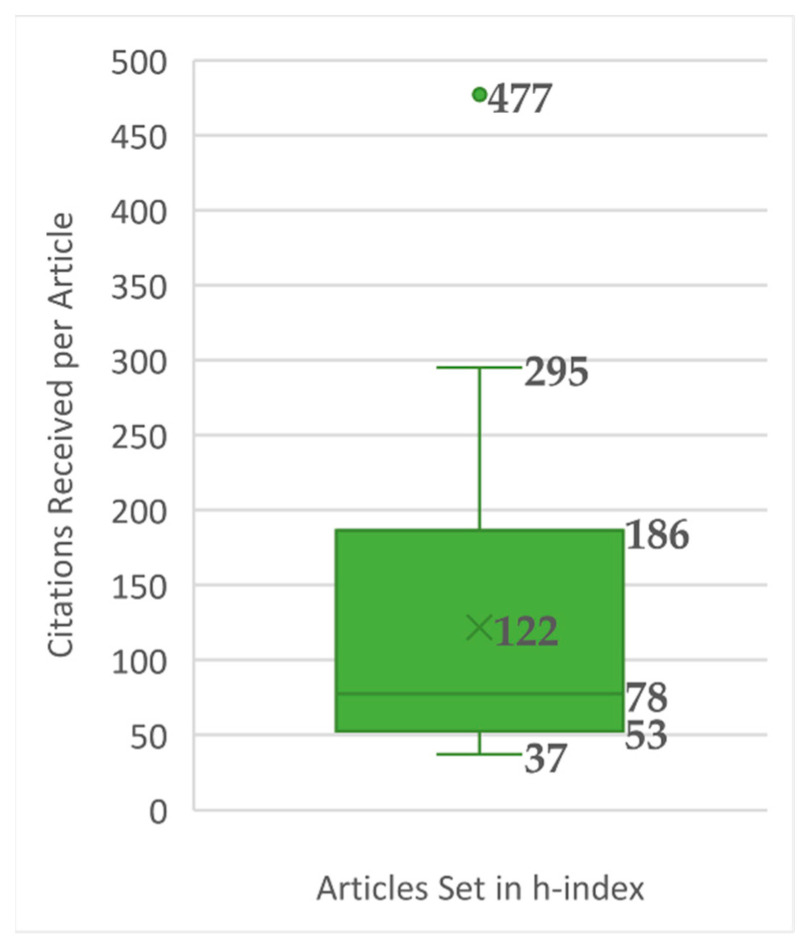
Box plot of citations received per article on coffee/caffeine and sport.

In terms of citation (metadata: Times Cited, WoS Core), we obtain, as a h-index or Hirsch index, a value of 34—thus, 34 articles cited 34 times or more, from 37 to 477 citations (see Appendix A). From this, we can already identify 126 low-impact articles, many of these without citations or with only one citation. However, within this set of 34 articles, there are notable differences in scientific evaluations expressed in citations. Figure 5 shows one article with atypical citations (477), and eight that are in the upper quartile, with between 186 and 295, highlighting nine articles that are immensely valued by the scientific community, above the mean of 122 and the median of 78 citations per article.

**Figure 6:**
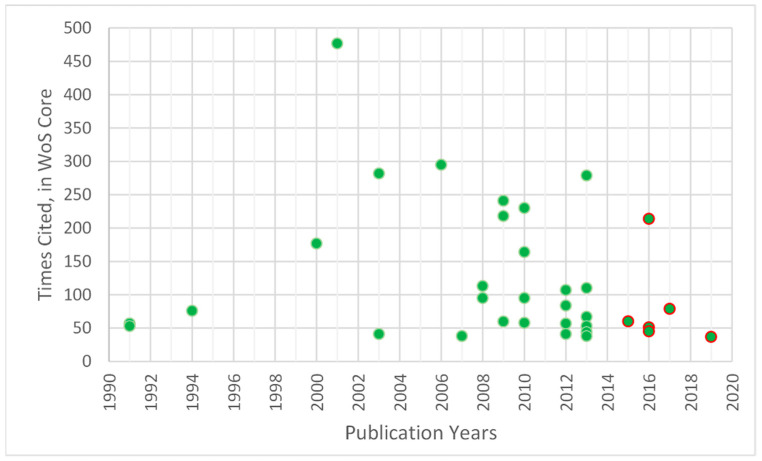
Citations received per article on coffee/caffeine and sport by publication year.

Figure 6 represents the citations achieved in terms of year of publication, among which six contemporary articles (2014–2020) stand out within the h-index, but it also allows us to identify the classics.

**Figure 7:**
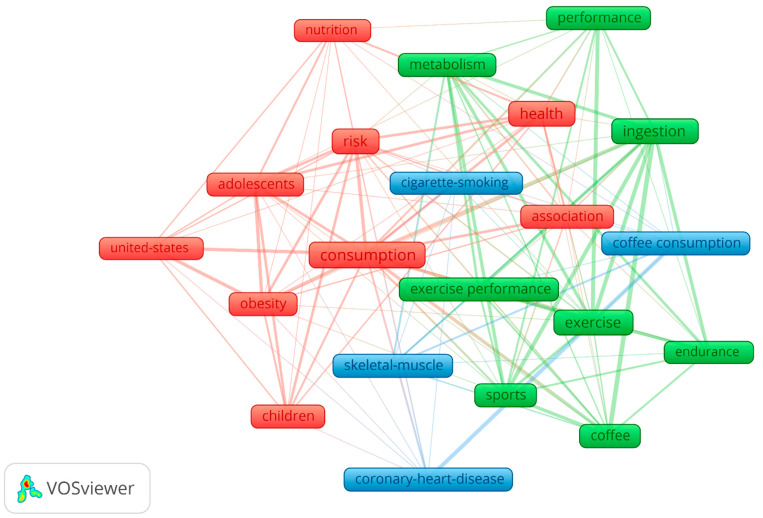
Thematic author keywords graph.

Finally, according to Zipf’s Law, three thematic clusters are identified that evince semantic differences, as represented in Figure 7: the first one centered on health and coffee consumption, associated with nutrition, risk, and obesity (red color); the second on metabolism and sport, associated with coffee intake, exercise, endurance, performance, and exercise performance (green color); and lastly, on coffee consumption and risk factors, associated with skeletal muscle, cigarette-smoking, and coronary heart disease (blue color).

**Figure 8:**
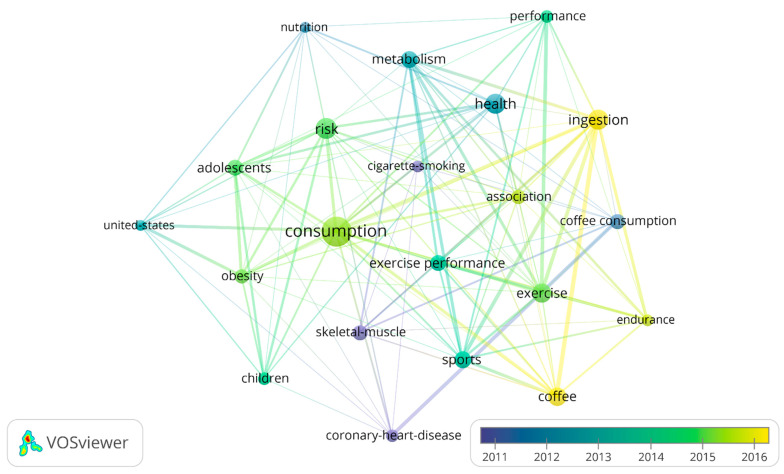
Temporary author keywords graph.

**Figure 9:**
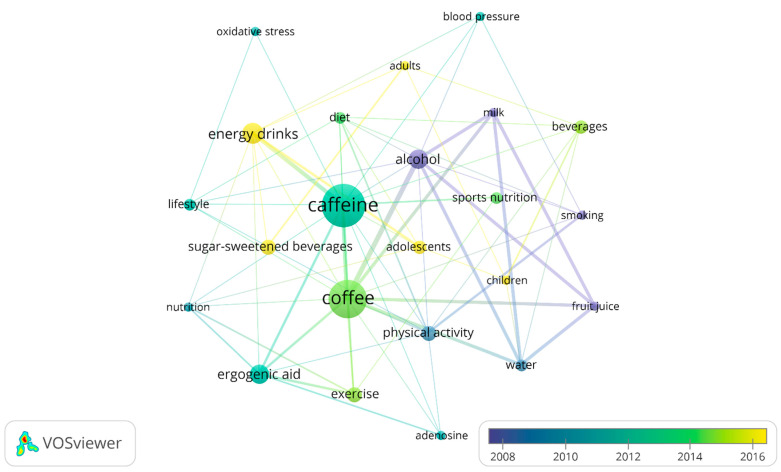
Temporary keywords plus graph.

**Figure 10:**
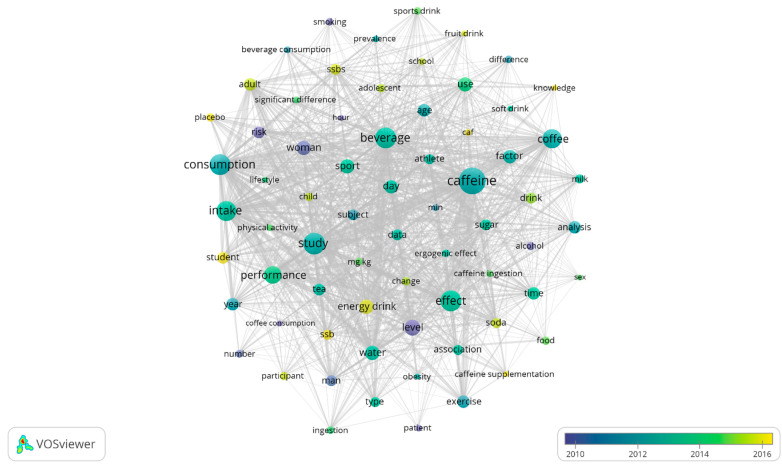
Temporary key terms graph.

However, a more interesting result is represented in Figure 8, Figure 9 and Figure 10, on the temporal evolution of keywords and key terms, showing an alternative trend to understand the role and effects of coffee and caffeine in sport. Thus, Figure 8 represents the temporal evolution of the 23 outstanding author keywords with four or more occurrences out of a total of 491 author keywords, showing the topics of the blue cluster as the oldest published on average (skeletal muscle, cigarette-smoking, and coronary heart disease), those of the red cluster (health and coffee consumption) with medium antiquity, and those of the green cluster (coffee and sport/exercise), with some topics of this last group being the most recent (coffee intake and endurance).

Figure 9 represents the temporal evolution of the 21 outstanding keywords with eight or more occurrences out of a total of 636 keywords plus. It is highlighted that, in 2014 (average publication date), concepts such as exercise, beverages, and diet (in light green) suddenly appear. Recent trends include studies focusing on energy drinks and sugar-sweetened beverages (ssb), colored in yellow.

Finally, the text data map for the key terms in Figure 10 results in 5161 terms extracted with VOSviewer ((Root Square (5161) ≈72), analyzing 69 key terms with 17 or more occurrences. This reinforces the temporal evolutions of the themes represented in the graphs of Figure 8 and Figure 9.

## 4. Discussion

In terms of bibliometric studies in nutrition, our study provides several advantages, such as the coverage of multiple journals, including the calculation of Bradford zones, unlike other bibliometrics in nutrition that focus on a single journal [41,47,64,65], and geographical coverage at a global level and not restricted to a specific country [65]. We also identified other bibliometric articles referring to specific nutritional topics [37,38,40,66] and others on sport and nutrition [46], but no specific bibliometrics relating coffee/caffeine to sport; therefore, this article is a novelty in this topic.

From a methodological standpoint, our study allows us to identify themes that refresh the ideas of some highly cited review articles presenting studies relating coffee/caffeine to sport [6,23,67,68]. Our study also provides greater coverage of the articles analyzed (larger article dataset size) than other recent review studies [17,25,26], also analyzing the relationships between the selected articles [35]. However, we recognize that this type of limited review study [17,25,26] tends to favor the specificity of the articles selected for analysis.

Our article highlights the contemporary trend of an alternative vision regarding the beneficial use of caffeine and coffee in sports performance, through the evidence of ergogenic studies on the increase in energy in athletes, when administered in adequate dosages in different formats of capsules, coffee, and sports drinks, including gum, bars, and gels [12].

Despite the possible ergogenic benefits reported in different studies, there are prestigious institutions that advise against the consumption of energy drinks in minors (The American Academy of Pediatrics, American Medical Association, etc.) [69] due to the number of negative effects of excessive caffeine intake [70]. Similarly, excessive caffeine intake could occur in adults for a purpose other than improving athletic performance, that is, the need to increase work productivity for fear of losing one’s job. In this sense, public health institutions should promote responsible consumption among potential consumers, whether they are adolescents, athletes, workers, or unemployed people, since these are addictive products that normally generate a high dependence among consumers.

## 5. Conclusions

An exponential growth of coffee/caffeine studies throughout the period1999–2020 is evidenced based on the 160 extracted articles (1938–2021). This shows a growing interest from the scientific community in the study of this topic, and determines a contemporary period of publications between 2014 and 2020, which comprises half of the articles published in the study period. As for the reference sources, out of a total of 117 journals, the following 12 comprise 30% of the publications: J. Strength Cond. Res. Sci. Sports Exerc., Sports Med., Am. J. Health Promot., Nutrients, BMC Public Health, J. Int. Soc. Sport Nutr., Int. J. Sports Med., Ann. Nutr. Metab., J. Acad. Nutr. Diet., Int. J. Sport Nutr. Exerc. Metab., and Am. J. Clin. Nutr.

At the author level, out of 667, only 52 have two or more publications in the topic studied, presenting a high fragmentation at the individual and institutional levels, which sets precedents of a competitive interaction (by structural equivalence). The USA is a true hegemony of scientific production in this topic, contributing 41% (65/160) of the articles, although the number of articles that exceed the h-index barrier within the extracted set is only 34 (21% of the total of 160), of which only six are recent or contemporary.

In its discourse, the set of articles evolves from coffee consumption and risk factors to studies on health and coffee consumption, and metabolism and sport correlated with coffee intake, adding energy drinks and sugar-sweetened beverages as specific topics in recent years.

## Figures and Tables

**Table 1:** Publications trends on coffee/caffeine and sport for core journals between 2001 and 2020.

Journals	2001–2010	2011–2020	Total
J. Strength Cond. Res.	3	3	6
Med. Sci. Sports Exerc.	3	2	5
Sports Med.	2	2	4
Am. J. Health Promot.	0	4	4
Nutrients	0	4	4
BMC Public Health	0	4	4
J. Int. Soc. Sport Nutr.	2	2	4
Int. J. Sports Med.	3	0	3
Ann. Nutr. Metab.	1	2	3
J. Acad. Nutr. Diet.	0	3	3
Int. J. Sport Nutr. Exerc. Metab.	0	3	3
Am. J. Clin. Nutr.	1	2	3
Total	15	31	46

## Data Availability

The analyzed dataset has been included in the Supplementary Materials.

## Supplementary Materials

The following are available online at https://www.mdpi.com/article/10.3390/nu13093234/s1, Table S1: SoCCaS210804.

## Appendix A

This appendix shows the articles selected by the Hirsch index (h-index), indicating their UT (Unique WOS ID): WOS:000171172100002; WOS:000236073100003; WOS:000180803900017; WOS:000320909200022; WOS:000270758400002; WOS:000274832500001; WOS:000266685800010; WOS:000390502100019; WOS:000089039700004; WOS:000273558300038; WOS:000271317700047; WOS:000329297000005; WOS:000299408700016; WOS:000282188300007; WOS:000255039600010; WOS:000299860000011; WOS:000409453700008; WOS:A1994NY45800001; WOS:000321151700001; WOS:000351779000010; WOS:000263752200009; WOS:000287926400001; WOS:000311290500007; WOS:A1991FF38500005; WOS:000329399300001; WOS:A1991FQ81000003; WOS:000375787200003; WOS:000371650200007; WOS:000325193900006; WOS:000309110700018; WOS:000186319200005; WOS:000316242400012; WOS:000247053300029; WOS:000470842800004.
